# Savoring Interventions Increase Positive Emotions After a Social-Evaluative Hassle

**DOI:** 10.3389/fpsyg.2022.791040

**Published:** 2022-03-21

**Authors:** Jeffrey J. Klibert, Bradley R. Sturz, Kayla LeLeux-LaBarge, Arthur Hatton, K. Bryant Smalley, Jacob C. Warren

**Affiliations:** ^1^Department of Psychology, Georgia Southern University, Statesboro, GA, United States; ^2^Center for Rural Health and Health Disparities, Mercer University School of Medicine, Macon, GA, United States

**Keywords:** savoring interventions, stress, social-evaluative hassles, savoring dimensions, positive emotions

## Abstract

Achieving a high quality of life is dependent upon how individuals face adversity. Positive psychological interventions are well-suited to support coping efforts; however, experimental research is limited. The purpose of the current research was to examine whether different savoring interventions could increase important coping resources (i.e., positive emotions) in response to a social-evaluative hassle. We completed an experimental mixed subject design study with a university student sample. All participants completed a hassle induction task and were then randomly assigned into different intervention groups. Positive emotion ratings were collected at three points in time (baseline, post-induction task, post-intervention). Results revealed a significant time x intervention interaction effect, such that individuals in the savoring the moment intervention reported higher levels of positive emotions (at post-intervention) compared to individuals assigned to the true control group, guided imagery control group, and savoring through reminiscence intervention. Such findings represent a significant extension to savoring theory and offer support for savoring the moment exercises as a primary prevention strategy to bolster effective responses to social-evaluative hassles.

## Introduction

### Rationale for Study

Positive psychological interventions (PPIs) are systematic attempts to foster a higher quality of life by activating and enhancing positive emotional states (e.g., joy, enthusiasm, pride) and character strengths [e.g., social intelligence, prudence, kindness (Seligman and Csikszentmihalyi, 2000; Quoidbach et al., 2015)]. Oftentimes, mental health clinicians employ PPIs through straightforward exercises including, but not limited to, guided imagery, narrative-based, and positive memory recall tasks (Bolier et al., 2013). Experimental findings pinpoint PPIs as effective mechanisms by which mental health practitioners can increase happiness and decrease depression (Seligman et al., 2005), reinforce social connectivity (Kerr et al., 2015), and minimize engagement in substance-use behaviors (Krentzman, 2013).

Interestingly, recent experimental evidence, stemming from randomized clinical trials (RCT), suggests PPIs are particularly effective in minimizing stress-related outcomes for different subpopulations of people. Mindfulness, resilience, character strength, and happiness training programs are just a few PPIs effective in minimizing stress-related outcomes (Ferrandez et al., 2021). As a collective group, numerous meta-analytic studies reveal relatively stable effect sizes for the impact of PPIs on stress-related outcomes (Carr et al., 2020; Hendriks et al., 2020; Ferrandez et al., 2021). Despite slight variation in selection criteria and RCT study quality, evidence consistently indicates PPIs demonstrate a moderate effect size in minimizing stress-related outcomes and these beneficial gains are maintained for at least 3 months (Carr et al., 2020). Despite these findings, there are still large gaps in evaluating *how* PPIs contribute to different well-being outcomes, especially in the context of stress and adversity (Fjorback et al., 2011; Quoidbach et al., 2015).

### Purpose of Study

Moving forward, identifying how PPIs foster mechanisms of action or internal resources to overcome stress and adversity is a key step in filling gaps in the positive psychological literature. Notably, emerging lines of examination suggests PPIs (e.g., Processing of Positive Memories Technique; Contractor et al., 2021) cultivate important psychological resources (i.e., positive emotions, adaptive thoughts, enhanced retrieval of positive memories) deemed useful in navigating through and growing from different facets of stress and adversity (Wong, 2011), yet little experimental research exists to corroborate this theoretical position. Guided by the tenets of the Broaden and Build model (Fredrickson, 2001), we examined whether different savoring strategies help individuals increase positive emotions after the experience of a social-evaluative hassle.

### Positive Psychological Practices to Manage Hassles

Hassles, episodic and unexpected strains on daily living, substantially affect the stability of a person’s cognitive-emotional functioning (Lazarus and Folkman, 1984; Serido et al., 2004). Research suggests the effects of hassles may provide a better explanation for the occurrence of debilitative psychological outcomes, when compared to negative life events (Lazarus and Folkman, 1984; Serido et al., 2004). Hassles are associated with a concerning set of psychological outcomes including relationship distress (Falconier et al., 2015), psychopathology (McIntosh et al., 2010), and reduced engagement in life-promoting behaviors (Jacob et al., 2014).

The emotional consequences of physiological strain are important in explaining the debilitative effects of hassles on different mental health outcomes. Research consistently demonstrates hassles contribute to diminished levels of positive emotions (e.g., Dowd et al., 2010). If these dysregulated emotional responses persist, individuals are more likely to exhibit vulnerability to a wide range of psychological difficulties (Sheppes et al., 2015). Given this pattern of findings, researchers are strongly encouraged to empirically examine mechanisms by which individuals can effectively regulate emotional responses to hassles (Robins et al., 2012; Cruess et al., 2015).

Currently, most positive coping programs are effective in reducing negative emotions through a wide array of interventions [e.g., loving kindness meditation (Feldman et al., 2010), mindfulness-based stress reduction (Keng et al., 2021) mindfulness smart phone applications (Economides et al., 2018), relaxation techniques (Hintz et al., 2015)]. Although the effects of positive coping programs on negative emotions are quite clear, there is mixed evidence to suggest mindfulness-based and relaxation-based stress management programs increase positive emotional outcomes. For instance, a handful of studies offer evidence for increases in positive emotions after completing different mindfulness (Economides et al., 2018) and meditation (Feldman et al., 2010) stress programs. However, other studies report decreases in positive emotions after completing short-term mindfulness-based and relaxation exercises (Lancaster et al., 2016). To explain these findings, researchers speculate some individuals may experience some mild discomfort because of a lack of familiarity with mindfulness or abstract components associated with brief mindfulness tasks, leading to small decrements in positive emotional outcomes (Lancaster et al., 2016). Considering these mixed findings, more research is needed to determine best pathways to increase and prolong positive emotional states after the experience of stress.

### Savoring, Positive Emotions, and Hassles

Increasing and sustaining positive emotions are fundamental components of resilience and thriving (Tugade and Fredrickson, 2007). Yet, when compared to negative emotions, research is only just emerging on how positive emotion interventions help bolster well-being in the face of adversity (Folkman, 2008). According to the Broaden and Build model (Fredrickson, 2001), positive emotions broaden momentary thought-action tendencies, which are characterized by an openness to diverse perspectives and ways of responding to stimuli. Essentially, positive emotions alter individuals’ cognitive processing style, making it more flexible, integrative, creative, considerate, and efficient in examining different options and opportunities for action. Moreover, broadened mindsets often build enduring personal resources (e.g., resilience, social support, positive re-appraisal skills), which individuals can use to manage future conflicts, challenges, and threats. These mindsets and resources are paramount in navigating hassles in a resilient manner (Tugade and Fredrickson, 2007). Specifically, marshaling positive emotions in the face of adversity increases resources needed to thrive (Zautra et al., 2005).

Unfortunately, the experience of hassles may restrict an individual’s ability to generate and sustain positive emotions, which in turn undermines the psychological benefits (e.g., creativity, optimism) associated with positive emotions. Interestingly, the experience of hassles gives rise to both negative and, paradoxically, positive emotions (Zautra et al., 2001, 2005), though negative emotions accelerate at a higher rate. Because of this skewed ratio, individuals who experience of hassles, particularly those containing two or more social-evaluative stressors, often report decreases in the production of creative ideas and solutions (Byron et al., 2010), which leaves them vulnerable to maladaptive health outcomes (Cheng et al., 2014; Waugh and Koster, 2015). Findings such as these underscore the need for researchers to develop and empirically validate programs designed to increase positive emotions in the face of hassles.

Savoring is an emotion regulation process whereby individuals generate, maintain, or enhance positive emotions through mindful appreciation of different life experiences (Bryant, 2003; Bryant and Veroff, 2007). Individuals can regulate positive emotions by employing savoring strategies associated with three different time dimensions: mentally reflecting upon past positive experiences (reminiscing), mindfully attending to positive experiences in the moment (moment), and contemplating positive experiences likely to occur in the future (anticipating; Bryant, 2003). Savoring is associated with several attributes known to promote a higher quality of life, especially resilience (Smith and Hanni, 2019). Moreover, research clearly highlights savoring as an important process in helping individuals maintain and increase positive emotions during minimal stress contexts (Jose et al., 2012).

However, evaluating the effects of savoring on stress is an emerging line of study and there is a stark need to understand how savoring affects positive emotions during adversity and challenge (Samios and Khatri, 2019). One reason for the lack of empirical investigation into savoring as a positive stress management strategy is the detrimental effects of stress; navigating through stress is cognitively taxing, making it difficult for individuals to notice, attend to, and savor positive emotions (Bryant and Smith, 2015). This position negates the idea of savoring as a central component in stress management efforts. Yet, meta-analytic and experimental studies highlight the beneficial effects of noticing, leveraging, and extending positive emotions in managing different types of stressors (Moskowitz et al., 2012; Zhang and Han, 2016). Considering how effective savoring strategies are at increasing the number and amplifying the intensity of positive emotions (Wilson and MacNamara, 2021), it seems warranted to evaluate savoring as an effective path by which people navigate through or overcome daily hassles.

Savoring interventions appear well suited to help individuals recover and extend positive emotions after a stressful event. Notably, savoring is part of the attentional deployment family of regulation tactics, those associated with selecting and modifying situations to increase positive emotions without changing the environment (Quoidbach et al., 2015). Such tactics often receive the most praise in terms of effectively regulating positive emotions across different contexts (Quoidbach et al., 2015). Second, savoring seems well suited to help individuals quickly access and increase positive emotions to initiate resilience-based processes during and after a social-evaluative hassle. Within the prevailing literature, the process of savoring generally coincides with effective efforts to cope with episodic stressors and hassles (Bryant and Smith, 2015). In fact, some researchers label savoring as a meaning-focused coping response, a method where individuals attend to positive events and emotions as a way to offset the debilitative effects of stressful life events (Folkman, 2008).

This emerging position is supported by a handful of studies. Specifically, coping with loss or managing challenging circumstances may paradoxically increase awareness and activation of positive attributes and strengths for individuals to savor. For example, a large proportion of caregivers are able to identify and celebrate unique aspects of their service duties amidst continuous hassles (Moskowitz et al., 1996). Specifically, caregivers demonstrate the capacity to savor positive experiences grounded in friendship and relish life’s small delights (e.g., a sunny day), leading to increases in positive emotional functioning. Other studies suggest savoring small blessings and positive experiences during the course of episodic hassles increases positive emotions, which then can be used to bolster effective coping outcomes (Folkman and Moskowitz, 2000). Finally, the use of savoring through behavioral expressions (i.e., smiling, laughing) while mourning the death of a partner at 6 months post-loss reduces a wide range of stress and grief-specific symptoms (Bonanno and Keltner, 1997). Similarly, savoring symbolic memories and sharing positive experiences with others after the loss of a loved-one (Samios and Khatri, 2019) may help individuals change the meaning behind their experience of stress and grief and provide some temporary relief in the form of peaceful and warm emotions.

### Current Study

Moving forward, experimental designs are needed to clarify if and how savoring contributes to positive coping efforts. Specifically, it is important to identify whether savoring can generate positive emotions in the face of social-evaluative hassles, events that threaten social esteem and status (Byron et al., 2010). To date, studies highlight savoring as an effective mechanism by which individuals can generate, maintain, and enhance positive emotions (e.g., Hurley and Kwon, 2013). However, most of these studies examine the effects of savoring in the context of positive life events. No known study has experimentally examined the causal link from savoring to positive emotions in the context of social evaluative hassles.

In addition, it is important to examine the effectiveness of different savoring strategies in increasing positive emotions after a hassle. To date, research is mixed regarding whether specific savoring strategies are more powerful in eliciting positive psychological outcomes. On one hand, research suggests practicing diverse savoring strategies is the best way to maximize well-being (Quoidbach et al., 2010); however, an emerging set of studies also highlight savoring in the present moment as more beneficial than savoring through the past (reminiscing) and future (anticipation). Time-foci interventions trigger different mechanisms for increasing positive outcomes (Wellenzohn et al., 2016). Differential time-foci effects appear particularly influential in short-term interval studies, although past-oriented interventions may be less effective in increasing positive emotions (Quoidbach et al., 2015). Taken as a whole, these findings highlight the potential benefits of using present-focused savoring interventions (savoring the moment) over past (savoring through reminiscence) and future-based (savoring through anticipation) interventions in eliciting short-term increases in positive emotions.

Given these findings, the purpose of the current study was to experimentally examine the differential effects of savoring dimension interventions (anticipation, moment, reminiscent) on positive emotions after a social-evaluative hassle. By evaluating savoring in this context, we hope to provide an empirical foundation by which a unique set of positive psychological skills can enhance situation-based coping efforts and resilience development. In light of applicable theory and empirical work, we hypothesized that individuals participating in a savoring intervention after experiencing a social-evaluative hassle would report higher levels of positive emotions compared to individuals assigned to the control conditions. In addition, we predicted this effect would be strongest for individuals assigned to the savoring the moment intervention.

## Materials and Methods

### Participants

The sample consisted of 145 students enrolled in a large southeastern university in the United States (US). In terms of inclusionary criteria, participants were required to be 18 years of age, which is the youngest age by which US citizens can provide consent to participate in research activities in the state in which the study took place. In addition, in order to ensure data quality, we included inclusionary criteria based on deviant response patterns. Specifically, to be considered in the final sample, participants needed to answer all validity check questions correctly and complete at least 90% of the survey items. All participants met these data quality criteria and were subsequently included in the final sample.

The sample included 107 (73.8%) women and 38 men (26.2%). Participants ranged in age from 18 to 25, with an average age of 19.35 (*SD* = 1.41) years. Participants self-identified as White/European American (*n* = 88, 60.7%), Black/African American (*n* = 38, 26.2%), multiethnic (*n* = 14, 8%), Mexican American/LatinX (*n* = 4, 2.8%), and Asian American (*n* = 2, 1.4%). In terms of academic information, a large majority of participants (*n* = 117, 80.6%) reported a desire to pursue a degree in psychology, with other students pursing degrees in other social science (e.g., sociology, criminal justice) and health-related (e.g., kinesiology, nursing) fields. Also, participants were dispersed across different years of their academic experience. Notably, participants identified as first-year students (*n* = 47, 32.4%), second-year students (*n* = 62, 42.8%), third-year students (*n* = 28, 19.3%), and fourth-year students (*n* = 8, 5.5%). Participants received course credit and/or extra credit for participating.

### Procedure

The university’s Institutional Review Board approved the study in advance of data collection. Data were collected in a secluded lab space containing a comfortable sofa and wall mounted video/audio equipment. Upon arrival, participants read and signed an informed consent sheet. They were told the study consisted of several self-report, verbal, and guided imagery tasks. Once participants decided to volunteer for the study, they completed the first administration of the Positive and Negative Affect Scale – Positive Affect (PANAS-PA; see below). Physiological biosensors were then clipped onto the participants’ fingers (see below). After a two-minute adaption period, all participants completed the hassle induction task (see below). Following the completion of the hassle induction task, participants completed the second administration of the PANAS-PA and were randomly assigned to one of five intervention groups. Completion time for each intervention was 15 min. The final administration of the PANAS-PA was given after participants completed the intervention tasks. Finally, given the nature of the study’s procedures, all participants were actively debriefed before leaving. As part of the active debriefing process, participants were given a list of free to low cost resources and engaged in a short stream of activity exercise effective in reducing minimal to moderate feelings of distress (Wilson and DuFrene, 2009). Participants completed all tasks in approximately 65 min.

### Experimental Design

The study employed an experimental two-way mixed (between and within) subject design to evaluate variation in positive emotions scores. Considering the need to evaluate savoring practices in the face of stressor, especially with non-cross-sectional and correlational designs (Samios and Khatri, 2019), we chose to evaluate our hypotheses using an experimental framework. The underpinning of our design stems from previous experimental work in the positive psychology literature. Notably, given the preliminary nature of the research and the short-lived nature of emotions (Levenson, 2014), we employed a short-wave design consistent with like studies (Chen et al., 2021). Our design also mirrors work from emotion recovery and resilience researchers, who commonly induce negative emotions through a wide range of induction tasks to better determine how different positive psychological interventions can improve positive emotional outcomes (Fredrickson and Levenson, 1998; Tugade and Fredrickson, 2004). We slightly altered this framework to induce stress before manipulating savoring interventions to better understand variation in positive emotion scores. In terms of the stress induction task, we used a social stressor commonly employed in emotion recovery studies (Leger and Charles, 2020) and measured participants’ cardiovascular arousal using multiple types of biosensors, consistent with best practices in stress-based experimental studies (Palumbo et al., 2017).

The hassle induction task employed was an adapted version of the Trier Social Stress Test (TSST; Kirschbaum et al., 1993), a common and effective means of eliciting moderate elevations in stress, consistent with the experience of a social-evaluative hassle (e.g., Tugade and Fredrickson, 2004). Participants were asked to prepare for a three-minute speech on an unrevealed topic. Then, participants were left alone for two minutes. When the experimenter returned, participants were informed of the theme of the speech: “Why I am an attractive person?” The participants were also informed their speech may be video recorded and recorded speeches may be shown to age-based peers in another study for evaluation. Then the experimenter left the participants alone for another 2 min. Once the experimenter returned, participants were asked to look into the video camera and give their three-minute speech. Despite the presence of the video camera, no speeches were videotaped.

### Interventions

Participants were randomly assigned to one of five groups: true control (*n* = 30), guided imagery control (*n* = 28), savoring through reminiscence (*n* = 27), savoring through anticipation (*n* = 30), or savoring the moment (*n* = 30). Group names reflected the nature and content of the activity immediately following the hassle induction and details of each intervention are outlined below. Each savoring intervention was targeted to the experience of joy as a means reduce threats to internal validity. Joy was chosen as the targeted emotional experience because aspects of it are well-captured under our identified measure of positive emotions (PANAS-PA). In addition, joy often produces a quieting functioning, a mechanism to minimize the negative effects and maximize the positive effects associated with social-evaluative hassles (Tugade et al., 2014).

#### True Control

Participants randomly assigned to the true control condition were not given a structured task to complete. The experimenter informed participants she needed to complete a few tasks important to the study and they should sit and process their experience. The purpose of the true control intervention was to simulate natural hassle processing without the aid of a structured exercise. In total, participants were left undisturbed for 15 min.

#### Guided Imagery Control

Participants randomly assigned to the guided imagery control intervention imagined taking a trip to the grocery store. The experimenter asked participants to find a comfortable position and close their eyes. Then, the experimenter used a script to guide participants through the imagery exercise. The script asked participants to cognitively process the acts of making a grocery list, driving, parking, and selecting needed groceries. A trip to the grocery store was chosen as the theme for the guided imagery exercise because of its common and neutral nature. Employing a guided imagery control is important as some evidence indicates even unremarkable interventions with calm and interactive features may affect relaxation and affective-control efforts (Leviton and Leviton, 2004; Trakhtenberg, 2008). By employing a guided imagery control, we attempted to control for basic intervention processes, which strengthens our ability to determine if savoring interventions positively impact positive emotional states after the experience of a social-evaluative hassle.

#### Savoring Through Reminiscence

Participants randomly assigned to the savoring through reminiscence intervention completed a memory-building exercise consistent with the practices outlined by Bryant and Veroff (2007, pg. 93). Participants were asked to:

“Search for a moment where you’ve found sweetness and joy in your life. I’d like you to recall a memory when you felt really alive. When all struggle from the hassles of daily life just paused for a moment… a moment completely without effort… a moment where you knew yourself, and knew you belonged. It could be something recent or something in the past. Try to call to mind just one memory. It can be a really powerful time or it can be something really simple, just recall a time when you felt joy. See if you can just be there in that memory, just allowing yourself to fall back into that moment of sweetness… lingering just briefly. When re-imagining yourself in this memory, I want you think about why it is important in terms of joy. Recall the memory from the very beginning to the end. Reflect on each person, feeling, and event that contributed to your sense of joy. I would like you to stay within this memory for the next few minutes.”

In this guided imagery exercise, participants were encouraged to reflect on unique features of their experience (e.g., personal strengths, interpersonal connections, thoughts) accentuating feelings of sweetness and joy. In total, participants spent 15 min completing the activity.

#### Savoring Through Anticipation

Participants randomly assigned to the savoring through anticipation intervention engaged in a planning for a positive event exercise (Bryant and Veroff, 2007, pgs. 120–121). Participants developed an itinerary for a weeklong vacation to a desired destination. Specifically, participants were instructed to:

“Imagine what it would be like to plan the vacation of your dreams. When ready, identify potential destinations and explore what your time there may be like. Imagine what you will hear, see, smell, taste, and feel. Think about who might join you on this vacation and how their presence may contribute to your sense of joy and excitement. In addition, think about what types of activities you might want to engage in while on your vacation and how they might enhance the fun and joy you hope to experience. Finally, think about how this vacation might surpass other experienced vacations in terms of fun, pleasure, and joy. Please imagine yourself in this scenario.”

In total, participants reflected on aspects of their planned vacation for 15 min.

#### Savoring the Moment

Participants randomly assigned to the savoring the moment intervention completed a heightened focus exercise (Bryant and Veroff, 2007, pgs. 116–119). Participants were asked to mentally review their emotions and how their emotions contribute to a good day. Then, participants were given a one page, strength-based passage, “*Your forces and how to use them*” written by Christian D. Larson. The passage consists of 24 lines broken into 6 stanzas, which includes themes of joy and optimism. With the passage in hand, participants were instructed to:

“Slowly savor every word and line of the passage deliberately and carefully. Let your mind pause, linger, and wonder over the meaningfulness of the passage. Read the words and lines over and over again to really let the feelings sink in. Again, take your time, be aware of the feelings that the words evoke, and savor the experience of those feelings.”

Participants were also asked to specifically reflect on how three of the more joyful lines of the passage (e.g., “*look at the sunny side of everything and make your optimism come true”*) strengthened emotions within them in the current moment. These lines were chosen to enhance the awareness and activation of savoring the moment processing. After 15 min, the experimenter ended the focus exercise.

### Measures

#### Cardiovascular Assessments

At the beginning of the experiment, non-invasive biosensors were placed on the fingers of each participant’s non-dominant hand to measure two components of cardiovascular activity: *heart rate (HR) and galvanic skin response (GSR)*. These measures were employed to evaluate whether the hassle induction task produced the intended physiological effect. HR is a common estimate of the heart’s electrical activity. Elevations in HR are expected when an individual is exposed to arousing stimuli. One HR electrode was clipped onto the participants’ ring finger and fluctuations in HR were measured in beats per minute (bpm). GSR is a measure of skin conductance, a process under the control of the sympathetic nervous system. During periods of arousal, individuals experience greater elevations in skin conductance (Villarejo et al., 2012). Two GSR electrodes were clipped onto the participants’ index and middle fingers and variation in skin conductance was measured in Micro Siemens (μS) units. Both measures of cardiovascular activity were continuously recorded throughout the duration of the study.

#### Positive Emotions

At three points during the study (beginning, post-hassle-induction, post-intervention) participants self-reported on positive emotions using the PANAS-PA (Watson et al., 1988) survey. The PANAS-PA is a 10-item measure of positive feelings resulting from positive experiences in one’s environment. The PANAS-PA assesses for state and trait indices of positive emotions. In the current study, participants rated the extent they felt interested, excited, strong, enthusiastic, alert, inspired, determined, attentive, proud and active in the current moment. The PANAS-PA is a commonly used measure of positive emotions in experimental studies evaluating the effects of brief positive psychological interventions on well-being outcomes (Feldman et al., 2010). The scale is one of the few measures effective in capturing short-term variation in different positive emotional states (Rogatko, 2009). Moreover, the scale is brief. This is an important consideration as longer surveys may dilute the intended effects of an intervention/manipulation while participants move in-between different tasks.

Participants were asked to rate the degree to which they experienced each dimension of positive emotion on a 100-point scale from 1 (*Very Slightly/Not at All*) to 100 (*Extremely*). The 100-point rating scale is an adaption from the original assessment, which used a 5-point scale. This adaptation was made in light of specific trends in college student responding to positive psychological surveys. Specifically, in some student samples responses to positive psychological surveys tend to be high, with a number of respondents reporting near-maximum or maximum scores (Klibert et al., 2019). This pattern of responding is consistent with the presence of a ceiling effect, which reduces the true range of scores, underestimates variability, and negatively affects estimates of reliability and validity for a specific measure (Uttl, 2005). In order to maximize variability in responses, we choose to adapt the rating scale. We adapted the scale in a manner that is consistent with clinical recommendations in estimating mood (e.g., Subjective Units of Distress Scale; Franklin and Foa, 2014). A total positive emotions score was calculated by summing individuals’ responses on each dimension. Total scores ranged from 10 to 1,000 with higher scores reflecting greater positive emotional functioning. The PANAS-PA demonstrates solid internal consistency (*α* = 0.89) and convergent validity with theoretically-related constructs (Rogatko, 2009). In the current study, the PANAS-PA demonstrated excellent internal consistency across different administrations (*α* = 0.87–0.92).

## Results

The current study evaluated variation in cardiovascular activity (HR and GSR) at two points (Time 1 and Time 2). The study also evaluated variation in positive emotion ratings at three points [baseline (Time 1), post-induction (Time 2), post-intervention (Time 3)]. Mean and standard deviations for HR, GSR, and positive emotion scores across time points and intervention groups are depicted in Table 1.

**Table 1:** Means and standard deviation scores for heart rate (HR), galvanic skin response (GSR), and positive emotions (PE) across time points and intervention groups.

Intervention group	Time 1			Time 2			Time 3		
	HR	GSR	PE	HR	GSR	PE	HR	GSR	PE
True Control									
Mean	75.929	28901.607	499.9	87.6897	44514.759	429.967	77.966	33341.9655	381.6
SD	15.981	8000.719	179.378	20.633	58721.919	176.389	15.661	5868.725	211.422
Guided Imagery Control									
Mean	81.308	31633.539	472.143	87.222	36110.407	418.454	77.889	35196.629	471.5
SD	20.894	6343.531	187.927	30.419	5021.807	241.324	20.554	5078.068	214.023
Savoring Reminiscence Intervention									
Mean	78.615	27030.039	489.222	75.52	34857.44	426.741	75.72	33819.04	495.852
SD	21.733	8257.114	178.191	21.608	6003.008	186.626	23.596	5277.638	230.439
Savoring Anticipation Intervention									
Mean	80.8	30,548	515.4	88.067	35877.233	462.067	76.9	35447.433	584.3
SD	15.829	7401.949	198.401	26.367	4738.523	233.345	19.148	4639.118	242.174
Savoring Moment Intervention									
Mean	74	28472.448	579.133	80.517	35415.897	510.967	69.8276	34239.931	633.167
SD	18.507	10619.94	190.328	24.423	6049.489	230.34	17.519	6079.216	214.915

### Sociodemographic Characteristics by Intervention Group

Participants were randomly assigned to five intervention groups (True Control, Guided Imagery Control, Savoring through Reminiscence, Savoring through Anticipation, Savoring the Moment). Table 2 provides a sociodemographic and academic breakdown of the participants randomly assigned into each group. Regarding gender, we evaluated whether groups significantly differed in terms of gender frequencies using a chi-square analysis. Results revealed no significant differences in gender frequencies for each group, *χ*^2^ (4) = 4.46, *p* = 0.35.

**Table 2:** Sociodemographic and academic class standing breakdown of participants by intervention group.

Demographic groups	Intervention groups				
	True Control (*n* = 30)	Guided Imagery Control (*n* = 28)	Savoring through Reminiscence (*n* = 27)	Savoring through Anticipation (*n* = 30)	Savoring the Moment (*n* = 30)
**Gender**					
Women	25 (83.3%)	21 (75%)	17 (63%)	20 (66.7%)	24 (80%)
Men	5 (16.7%)	7 (25%)	10 (37%)	10 (33.3%)	6 (20%)
**Ethnicity**					
White/European American	18 (60%)	20 (71.4%)	14 (51.9%)	19 (63.3%)	17 (56.7%)
Black/African American	11 (36.7%)	4 (14.3%)	9 (33.3%)	8 (26.7%)	6 (20%)
Multiethnic	1 (3.3%)	2 (7.1%)	2 (7.4%)	3 (10%)	4 (13.3%)
Mexican American/LatinX	0 (0%)	0 (0%)	1 (3.7%)	0 (0%)	3 (10%)
Asian American	0 (0%)	2 (7.1%)	0 (0%)	0 (0%)	0 (0%)
**Academic Cohort**					
1st Year Student	11 (36.7%)	13 (46.4%)	7 (25.9%)	8 (26.7%)	8 (26.7%)
2nd Year Student	12 (40%)	9 (32.1%)	10 (37%)	18 (60%)	13 (43.3%)
3rd Year Student	4 (13.3%)	5 (17.9%)	8 (29.6%)	4 (13.3%)	7 (23.3%)
4th Year Student	3 (10%)	1 (3.6%)	2 (7.4%)	0 (0%)	2 (6.7%)

### Heart Rate

**Figure 1:**
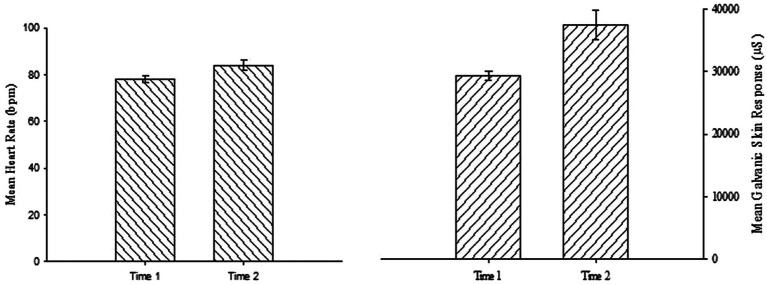
Mean heart rate and galvanic skin response score across Time. Errors bars represent standard errors of the mean.

As shown in Figure 1 (left panel), mean HR increased after the hassle induction for all interventions. These results were confirmed with a two-way mixed analysis of variance (ANOVA) on HR with Intervention (true control, guided imagery control, savoring through anticipation, savoring through reminiscence, savoring the moment) and Time (time 1, time 2) as factors which revealed only a main effect of Time, *F*(1, 133) = 7.4, *p* = 0.008, $\eta_{p}^{2}$ = 0.05. Neither the effect of Intervention, *F*(4, 133) = 1.1, *p* = 0.36, nor the interaction, *F*(4, 133) = 1.2, *p* = 0.31, were significant. These findings indicate that HR increased from Time 1 to Time 2 consistent with the intended effect and no pre-group intervention differences were detected for HR at baseline.

### Galvanic Skin Response

As shown in Figure 1 (right panel), mean GSR increased after the hassle induction for all interventions. These results were confirmed with a two-way mixed ANOVA on GSR with Intervention (true control, guided imagery control, savoring through anticipation, savoring through reminiscence, savoring the moment) and Time (time 1, time 2) as factors which revealed only a main effect of Time, *F*(1, 133) = 12.87, *p* < 0.001, $\eta_{p}^{2}$ = 0.09. Neither the effect of Intervention, *F*(4, 133) = 0.61, *p* = 0.66, nor the interaction, *F*(4, 133) = 0.80, *p* = 0.53, were significant. These findings indicate that GSR increased from Time 1 to Time 2 consistent with the intended effect and no pre-group intervention differences were detected for GSR at baseline. Taken together, these findings suggest the social-evaluative hassle was successful in inducing the desired cardiovascular arousal, which significantly differed from baseline reports.

### Positive Emotions

**Figure 2:**
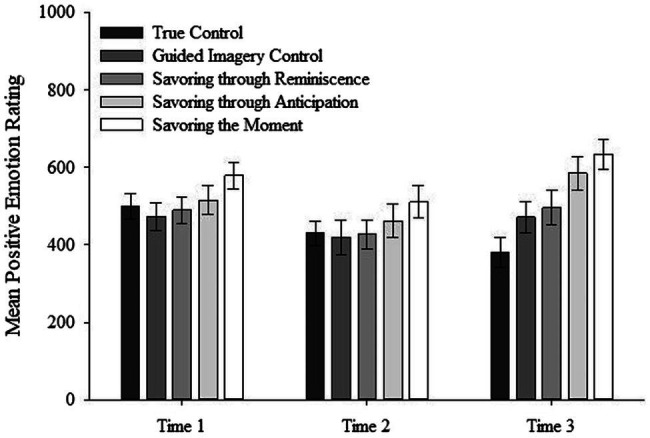
Interaction effect between time and intervention on positive emotions. Errors bars represent standard errors of the mean.

As shown in Figure 2, ratings of positive emotions did not differ between interventions for Time 1 or Time 2, but did differ between Interventions at Time 3. These results were confirmed by a two-way mixed ANOVA on positive emotions ratings with Intervention (true control, guided imagery control, savoring through anticipation, savoring through reminiscence, savoring the moment) and Time (time 1, time 2, time 3) as factors which revealed a main effect of Intervention, *F*(4, 140) = 2.57, *p* = 0.04, $\eta_{p}^{2}$ = 0.07, a main effect of Time, *F*(2, 280) = 16.53, *p* < 0.001, $\eta_{p}^{2}$ = 0.12, and a significant Intervention x Time interaction, *F*(8, 280) = 4.53, *p* < 0.001, $\eta_{p}^{2}$ = 0.12. Based on effect size measures ( $\eta_{p}^{2}$ ), the interaction effect accounted for 16.6% of the differences in positive emotions scores. When the $\eta_{p}^{2}$ is converted to *Cohen’s f* the effect equals 0.45, which is considered large in size (Cohen, 1988).

To isolate the source of the interaction, we conducted separate one-way ANOVAs for each Time with Intervention as a factor. There were no differences in Interventions at Time 1, *F*(4, 140) = 1.42, *p* = 0.23. This suggests participants randomly assigned to different intervention groups reported comparable scores on positive emotions at baseline. There were also no differences in Interventions at Time 2, *F*(4, 140) = 0.92, *p* = 0.46. However, results did reveal a significant difference between Interventions at Time 3, *F*(4, 140) = 5.84, *p* < 0.001. Results of Fisher’s LSD *post hoc* tests are shown in Table 3. Results demonstrate a pattern of scores which highlights the beneficial effects of savoring, particularly savoring the moment, in generating higher levels of positive emotions post-intervention (time 3). Specifically, in response to a social-evaluative hassle, individuals in the savoring the moment group (*M* = 633.17) generated higher levels of positive emotions when compared to individuals in the true control group (*M* = 381.6), the guided imagery control group (*M* = 471.5), and the savoring through reminiscence group (*M* = 495.85).

**Table 3:** Fisher’s least significant differences *post -hoc* test at time 3.

	1	2	3	4	5
1. True Control	–	−89.9*^ns^*	−114.25*^ns^*	−202.7^**^	−251.57^***^
2. Imagery Control		–	−24.35*^ns^*	−112.8*^ns^*	−161.67^**^
3. Savoring Reminiscence			–	−88.45*^ns^*	−137.32^*^
4. Savoring Anticipation				–	−48.87*^ns^*
5. Savoring Moment					–

ns = not significant

* = *p* < 0.05

** = *p* < 0.01

*** = *p* < 0.001.

In addition, results highlight mixed evidence regarding the beneficial effects of savoring through anticipation on positive emotions. Notably, post-intervention (time 3) results reveal individuals who participated in the savoring through anticipation intervention (*M* = 584.3) reported higher levels of positive emotions compared to individuals assigned to the true control group (*M* = 381.6), but not individuals assigned to the guided imagery control group (*M* = 471.5). Individuals who completed the savoring through reminiscence intervention did not report higher positive emotion scores compared to individuals in the true control and the guided imagery control groups.

## Discussion

Recent evidence suggests PPIs are advantageous in terms of helping people thrive in the face of adversity (Wong, 2011); however, no studies, to date, offer experimental support for savoring as an effective method to increase positive emotions after a social-evaluative hassle. Guided by the tenets of positive emotional theories (e.g., Fredrickson, 2001; Zautra et al., 2005), we explored the impact of different savoring interventions in increasing positive emotions after a social-evaluative hassle. In our experimental study, all individuals experienced a social-evaluative hassle as indicated by increased HR and GSR following the hassle-induction activity. Importantly, individuals in the savoring the moment intervention reported elevated levels of positive emotions after experiencing a social-evaluative hassle when compared to individuals assigned to either of the control tasks. To our knowledge, this is the first study to examine the causal effects of savoring on positive emotions in the context of a hassle. Our findings demonstrate the flexibility by which savoring interventions can be employed. Of note, savoring is not only a means of capitalizing on positive life events, it may also be an important component, a meaning-focused coping response (Folkman, 2008), in how individuals increase positive emotions after a hassle.

Our findings also highlight differential effects by which savoring dimensions increase positive emotions in the face of social-evaluative hassles. Consistent with our expectation, savoring the moment appeared relatively superior in terms of increasing positive emotions. Individuals assigned to the savoring the moment task reported higher positive emotion scores when compared to individuals assigned to true control, guided imagery control, and savoring through reminiscence tasks; however, positive emotion scores did not differ between individuals assigned to the savoring the moment versus the savoring through anticipation task, though the difference between the two groups’ scores (48.87) is trending in the expected direction.

Neither the savoring through anticipation nor the savoring through reminiscent task was able to consistently generate statistically higher levels of positive emotions compared to the control tasks. For instance, individuals who completed the savoring through anticipation intervention did report higher positive emotions scores when compared to individuals assigned to the true control group, but not to individuals assigned to the guided imagery control group. There are few clear reasons for this pattern of findings. It is possible that the effects of savoring through anticipation interventions may be smaller in size and our study was under-powered to such an extent, we could not detect these smaller effects. Future researchers should re-evaluate our findings with larger sample sizes to better determine if savoring through anticipation interventions are effective in producing higher positive emotion states after the experience of a social-evaluative hassle.

Delineated effects among the three savoring interventions are consistent with literature suggesting savoring in the present moment demonstrates stronger connections to positive psychological resources (Bryant, 2003); however, it is not completely understood why present moment strategies offer more practical benefits compared to other savoring techniques. One possible explanation for these differentiated effects is the manner by which forecasting (anticipation) and recall (reminiscent) guided imageries were constructed in our experiments. Imageability is a construct developed through the cognitive science field to describe the amount of contextual or perceptual detail needed to facilitate enriched memory recall of past events and projection of future events (Rasmussen and Berntsen, 2014). It is possible to manipulate the level of imageability through instruction (Williams et al., 1996). Specifically, recall and forecasting exercises that include more cued prompts are likely to facilitate enriched cognitive processing. For instance, when asking individuals to recall an event, it is important to instruct them to think about the following: (a) when the event occurred and in what context, (b) who was present during the event, and (c) what were salient feelings and thoughts experienced during the event. Including such cued prompts in guided imagery instructions may maximize the impact of memory recall and forecasting exercises on positive emotions. It is possible our anticipation and reminiscent interventions lacked enough cued prompts, which in turn mitigated their effects on positive emotions. Future research should consider the effects of imageability in maximizing the impact of anticipation and reminiscent strategies on positive emotions and other positive psychological outcomes.

The lack of a practically significant finding for the savoring through reminiscence intervention may also be explained by extreme variation in participant mood states. Notably, individuals who experience depression often report deficits in memory fluency and specificity, especially regarding the remembrance of positive autobiographical events (Hitchcock et al., 2020), making it difficult for these individuals to benefit from positive recall exercises. Moving forward, it is important for researchers to re-evaluate our findings, while using measures of depression as a covariate. Because we did not control for depressive symptoms, it is possible our research design could not adequately detect significant effects for our savoring through reminiscence and savoring through anticipation interventions.

In addition, the identified effects may be a result of differences in emotional experiences and qualities among the three savoring interventions. Although we made attempts to keep our savoring interventions parallel by orienting them to the experience of joy, it is possible that one intervention could have elicited a greater emotional range of experience. For instance, the passage component associated with our savoring the moment intervention may have strengthened greater emotions associated with pride and determination compared to the savoring through anticipation and savoring through reminiscence interventions. If this is the case, elicited emotional experiences may have moderated the degree to which savoring interventions impacted positive emotions. It is important for future research to consider the effects of emotional experience and qualities elicited by each savoring intervention to better differentiate whether savoring the moment interventions offer more benefits in generating positive emotions after a social-evaluative hassle.

Finally, these findings may simply highlight savoring the moment interventions as the most effective strategy to increase positive emotions after a social-evaluative hassle. Savoring the moment interventions rely heavily on the mindful experience of positive stimuli contained within a specific life event (Bryant and Veroff, 2007). On the surface, this appears antithetical when examined in the context of a hassle, as most hassles stimulate an overwhelming series of negative stimuli; however, research consistently demonstrates the availability of positive stimuli during the course of a stressful encounter (Zautra et al., 2005). Our findings provide preliminary support for the position that if individuals are able to quickly identify and sharpen focus on positive stimuli through the use of savoring the moment strategies (e.g., heightened focus, intensifying the moment), they are likely to exhibit increases in positive emotions after experiencing a social-evaluative hassle.

Overall, strength-based theories highlight the beneficial effects of employing PPIs in the context of challenge, adversity, and conflict. Specifically, PPIs have the capacity to increase positive emotions which broaden the mindset and build enduring coping resources needed to combat the negative effects of stress and hassles (e.g., Fredrickson, 2001). Our results provide support for these theories and offer preliminary evidence for the beneficial effects of savoring as a mechanism by which positive emotions can be increased in the face of a social-evaluative hassle. Moving forward, it is important for researchers to examine the effects of savoring the moment interventions against other, more empirically validated methods of coping with hassles. Specifically, it is critical to compare the effects of savoring the moment to known effective interventions (e.g., Progressive Muscle Relaxation). Such direct comparisons are essential to determine if savoring the moment interventions provide any additive benefits not accounted for by other empirically validated methods.

### Future Directions and Limitations

The current study offered an experimental perspective on how different savoring interventions increase positive emotions after a social-evaluative hassle. Although our study yielded interesting and promising findings, they are preliminary. Some methodological and sampling limitations need to be addressed before any conclusions drawn from the current research can be considered generalizable and critical to coping efforts. First, interpretations regarding the impact of savoring the moment should not extend beyond the scope of social-evaluative hassles. We did not assess for the effectiveness of different savoring interventions in increasing positive emotions after negative life events, trauma, or loss. Therefore, we can only suggest savoring the moment interventions may be helpful in producing important emotional resources in the context of relatively minor, episodic, and social forms of stress.

Second, because of the single post-intervention assessment nature of our design, we cannot infer whether the effects of different savoring interventions induce enduring levels of positive emotions. However, because the source of stress investigated was minor and episodic, the longer-term effect of savoring the moment was not germane to the core of our investigation. Studies investigating the longer-term benefit of savoring interventions against more chronic or long-lived forms of stress (e.g., trauma) are still needed.

Third, based on the findings offered, we cannot state that savoring interventions contribute to successful coping efforts. Instead, we can only state that savoring the moment can increase positive emotions in the face of social-evaluative hassles, which are important in the coping process, but alone may not account for complete coping success. In the future, it is important to determine whether savoring interventions can increase other important coping resources (e.g., coping self-efficacy, resilience) to better determine the impact of savoring on the entire coping process. In addition, it also important to determine how savoring interventions affect negative emotions and cardiovascular arousal. The intent of our study was to isolate the effects of savoring interventions on positive emotions after a social-evaluative hassle. Because of this focus, we did not measure whether savoring intervention could offset the effects of negative emotions or minimize cardiovascular arousal. It is important for future research to examine these lines to better facilitate theory on how savoring impacts stress recovery. Moreover, it is important to determine if the beneficial effects of savoring the moment interventions hold through these lines of inquiry.

Fourth, we sampled university students, which limits the generalizability of our findings to different subpopulations of people. Interestingly, research suggests savoring interventions, along with other PPIs, are most effective when employed with individuals who experience fewer positive life events (Hurley and Kwon, 2013). As a result, the use of a university sample may underestimate the effects of savoring in this context. It is important for researchers to validate our findings using diverse samples of individuals including clinical samples, individuals from lower SES backgrounds, and individuals who experience a larger amount of discrimination and oppression.

Fifth, the self-report nature of how we assessed positive emotions is susceptible to demand characteristics and social desirability concerns, although this effect theoretically was accounted for in the randomized design. Future research may want to consider evaluating the current research questions using observational and behavioral measures of positive emotions to increase the validity of our findings. For instance, instead of relying on self-report assessments, researchers may want to also consider using facial recognition programs to track facial expressions (e.g., smiling) specific to positive emotions in the context of different savoring interventions.

Sixth, because our savoring the moment intervention included a passage referencing a number of positive strengths, it may be useful to identify which of these elements is the strongest active ingredient in producing positive emotions. It may also be useful to generate and compare multiple savoring the moment interventions. This may lead to more focused or individually-tailored PPIs.

Finally, it may be fruitful to manipulate the experience of stress (high stress vs. low stress induction) instead of requesting all participants complete a social-evaluative hassle task. By manipulating stress, researchers may be able to determine if different savoring interventions moderate the effects of social-evaluative hassles on different emotional outcomes. Uncovering such effects may be important in determining whether or not savoring can buffer or undo the negative effects of stress on emotional well-being.

### General Conclusions and Practical Implications

Despite these limitations, the current research breaks new ground in several respects. This is the first study to find experimental support for the position that savoring the moment techniques are well suited to increase positive emotions after a social-evaluative hassle. In addition, there is some evidence to suggest brief savoring the moment exercises may be more effective in increasing positive emotions when compared to other savoring approaches. Taken as a whole, our findings highlight savoring the moment techniques as a promising primary prevention strategy to help manage episodic and unexpected hassle. From a practical standpoint, savoring the moment exercises can help individuals identify, focus on, and increase positive emotions that are already naturally occurring during hassles. Furthermore, these strategies are brief, easy to administer, and elicit heightened levels of enthusiasm and interest (Bryant and Veroff, 2007). Considering unresolved hassles are likely to decrease quality of life, employing savoring the moment techniques may be an important means of facilitating greater coping resources. We recommend mental health professionals develop outreach programs by which university and community members can learn about and practice employing savoring the moment techniques in the face of social-evaluative hassles.

## Data Availability Statement

The raw data supporting the conclusions of this article will be made available by the authors, without undue reservation.

## Ethics Statement

The studies involving human participants were reviewed and approved by Georgia Southern University – Institutional Review Board. The patients/participants provided their written informed consent to participate in this study.

## Author Contributions

JK, KL-L, AH, KS, and JW contributed to creation and design of the study. KL-L and AH took administer the procedures to participants. JK and BS were responsible for cleaning and analyzing the data. JK wrote the majority of the manuscript. BS contributed to the writing of the results and the development of the tables and figures. KL-L, AH, KS, and JW wrote pieces of the manuscript. All authors contributed to manuscript revision and approved the submitted version of the manuscript.

## Conflict of Interest

The authors declare that the research was conducted in the absence of any commercial or financial relationships that could be construed as a potential conflict of interest.

## Publisher’s Note

All claims expressed in this article are solely those of the authors and do not necessarily represent those of their affiliated organizations, or those of the publisher, the editors and the reviewers. Any product that may be evaluated in this article, or claim that may be made by its manufacturer, is not guaranteed or endorsed by the publisher.
